# Knocking down Sterol regulatory element binding protein 2 (SREBF2) inhibits the Serine Protease 8 (PRSS8) /sodium channel epithelial 1alpha subunit (SCNN1A) axis to reduce the cell proliferation, migration and epithelial-mesenchymal transformation of ovarian cancer

**DOI:** 10.1080/21655979.2021.1978615

**Published:** 2021-11-25

**Authors:** Chunyan Cai, Yumei Zhang, Xing Peng

**Affiliations:** Department of Gynaecology, The Affiliated Huaian No.1 People’s Hospital of Nanjing Medical University, Huai’an, China

**Keywords:** SREBF2, PRSS8, SCNN1A, ovarian cancer

## Abstract

The pathogenesis of ovarian cancer (OC) is complex. Serine Protease 8 (PRSS8) is a potential biomarker for early detection of OC. Multiple databases were used to predict the expression of PRSS8, Sterol regulatory element binding protein (SREBP) and sodium channel epithelial 1alpha subunit (SCNN1A) in OC patients and to detect the relationship among the three. The expressions of PRSS8, SREBF2, SCNN1A and related factors of the pathway were detected by RT-qPCR and Western blot. The cell transfection was used to overexpress or inhibit the expression of PRSS8 and SREBF2, so as to explore its mechanism. MTT assay and Colony formation assay were used to detect cell proliferation. The Transwell and Wound Healing assays were utilized to measure cell invasion and migration. We have further confirmed cell-level studies in animals. We found that PRSS8 expression was up-regulated in OC patients and cell lines. Knocking down PRSS8 reduced the proliferation, migration and epithelial-mesenchymal transition (EMT) of OC cells, which was realized by SREBF2 transcriptional regulation. Knocking down SREBF2 reduced PRSS8 and then inhibited the expression of SCNN1A, thus affecting the proliferation, migration and EMT of OC cells. These results also applied to animals experiments. In conclusion, SREBF2 activates the PRSS8/SCNN1A axis to accelerate cell proliferation, migration and EMT of OC.

## Introduction

Ovarian cancer (OC) is the most fatal gynecological tumor in women worldwide, which seriously affects the health and quality of life of patients [1]. However, due to the unnoticeable symptoms in the early stage of the disease, about 70% of OC patients are found in the late stage, missing the optimal treatment phase [2]. Therefore, finding therapeutic targets for OC and exploring the pathogenesis of OC are significantly helpful for the early detection and treatment of OC.

Serine Protease 8 (PRSS8) is currently identified as a potential biomarker for the early detection of OC [3]. In the occurrence and development of OC, the overexpression of ZNF217 promotes the immortality of tumor cells. After Knockdown of ZNF217 can inhibit the malignant progression of OC cells, together with decreased expression of PRSS8 [4]. Notably, from the above evidence, PRSS8 may be related to the OC development. However, the specific role and mechanism of PRSS8 in OC have not been reported so far.

The sterol regulatory element binding transcription factor 2 (SREBF2) was predicted to be a transcription factor of PRSS8 by bioinformatics. It was found that SREBF2 is one of the mediators of the tumor suppressor activity of miR-28-5p in prostatic carcinoma cells, and the inhibition of SREBF2 expression can significantly affect the progression of prostate tumors [5]. Downregulation of SREBP inhibits tumor growth and genesis by altering metabolism of colon cancer cells [6]. In addition, studies have shown that SREBP2 can promote cisplatin resistance in OC cells [7].

Therefore, we speculate that SREBF2 may play an influence on the OC proliferation, migration and ETM by regulating the PRSS8/SCNN1A axis, and thus we aimed to test this conjecture in this paper.

## Materials and methods

### Source of clinical samples

Plasma of healthy subjects and ovarian cancer patients were collected from Huai ‘an First People’s Hospital of Nanjing Medical University, with 5 patients in each group. The experiment was approved by the Ethics Committee of Huai ‘an First People’s Hospital Affiliated to Nanjing Medical University (the number of the approval: YX-2021-030-01), and all participants signed the written informed consent.

## Cell culture

A human normal ovarian epithelial cell line (IOSE-80) and ovarian cancer cell lines SKOV-3, OVCAR3, A547 and A2780 cells purchased from the ATCC (Manassas, VA, USA) were maintained in RPMI1640 supplemented with 10% fetal bovine serum (FBS) at 5% CO_2_ with 37 C, following the method previously reported by Chien et al [8].

## Western blot

RIPA (Beyotime Technology, Jiangsu, China) was used for protein extraction. Protein concentration was measured using a BCA protein assay kit (Beyotime Institute of Biotechnology). 40 µg protein were separated using SDS‐PAGE and transferred onto PVDF membranes, which were subsequently blocked with 5% nonfat milk and incubated with relevant primary antibodies at 4°C overnight. Horseradish peroxidase‐conjugated secondary antibodies were used to detect primary antibodies. The ECL detection system (Millipore, Billerica, MA, USA) was used to image protein bands and a semiquantitative analysis was conducted using ImageJ software. Primary antibodies included GAPDH (1:1,000, ab8226, Abcam, Cambridge, MA, USA), anti‐PRSS8 (1:1,000, ab185236, Abcam), anti‐E-cadherin (1:1,000, ab40772, Abcam), anti‐N-cadherin (1:500, ab76011, Abcam), anti‐Vimentin (1:1,000, ab925470, Abcam), anti‐Vimentin (1:1,000, ab925470, Abcam), anti‐SREBF2 (1:1,000, ab30682, Abcam), anti‐SCNN1A (1:1,000, ab214192, Abcam), anti‐SCNN1A (1:1,000, ab214192, Abcam), anti‐SCNN1A (1:1,000, ab214192, Abcam).

## Plasmid construction and transfection

The PRSS8 gene was knocked down using two different PRSS8 short hairpin (sh) RNAs (shPRSS8-1 and shPRSS8-2) with lentiviral expression vector GV 493. A negative control shRNA (sh-NC) was used to control for off‐target and nonspecific effects of shRNA treatment at a concentration of 20 nM. All above were purchased from Shanghai GenePharma Co., Ltd., using Lipofectamine® 2000 reagent (Invitrogen; Thermo Fisher Scientific, Inc.). For overexpressing SREBF2, SREBF2 overexpression plasmid was transfected into cells and blank control plasmid (GenePharma, Shanghai, China). Cell transfections were conducted using the Lipofectamine 2000 (Invitrogen) according to the manufactures’ instruction.

## RT-qPCR

Total RNA was extracted from cells or plasma using TRIZOL reagent (Invitrogen). RNA was reverse transcribed into cDNA using a PrimeScript RT Reagent Kit (Takara, Japan). qPCR was performed using QuantStudio™ Test Development Software (Thermo Scientific, USA) with SYBR Green qPCR Master Mix (Rox) (Roche, Mannheim, Germany). The cDNA was synthesized from total RNA (0.5 μg) at 48°C for 30 min and at 95°C for 10 min. The cDNA (0.7 μg) was subjected to polymerase chain reaction (PCR) by 45 cycles at 94°C for 45 seconds, 56°C for 30 seconds, and 72°C for 30 seconds. These primers used in quantitative reverse transcription-PCR were as follows::PRSS8 primer (forward: 5′- GATTACTCCGGTCGGGGAC-3′, reverse: 5′-ACGCCTTCATAGGTGATGCT-3′) and GAPDH primer (forward: 5′- GCACCGTCAAGGCTGAGAAC −3′, reverse: 5′- TGGTGAAGACGCCAGTGGA −3′).The data were analyzed using the 2^−ΔΔCT^ method [9].

## MTT

After the cells were transfected, The Vybrant® MTT Cell Proliferation Assay Kit (Invitrogen Thermo scientific, Carlsbad, CA, USA) was used according to the manufacturer’s protocol. At different time points (24, 48, and 72 h), 20 μl MTT (5 mg/ml) was added to each well and incubated for an additional 4 h at 37°C. Finally, dimethyl sulfoxide (DMSO, Sigma, USA) was added to stop the reaction, and the absorbance was detected using a microplate reader (Thermo Fisher Scientific, Inc.).

## Colony formation assay

Colony formation assay was used to evaluate the clonogenic capability of ovarian cancer cells. Cells at a density of 2 × 10^3^ were trypsinized in a single-cell suspension and seeded in 6-well dishes. Afterward, the cells were maintained in DMEM (Gibco) supplemented with 10% FBS (Sigma-Aldrich) for about 2 weeks. The visible colonies were fixed in 4% paraformaldehyde (Solarbio) for 4 h at 37 C and stained with 0.5% crystal violet (Beyotime Institute of Biotechnology, Haimen, China) for 2 h at 37 C. Colony numbers were counted under a light microscope (Olympus Corporation).

## Wounding healing

SKOV3 cells were cultured in 6-well at a density of 4 × 10^5^. When the cells grew to >90% confluence, a scratch was performed with a 200 µl pipette tip to make a linear wound in the central area. Then cells were washed with PBS, and migrated cells were counted under a phase-contrast microscope (magnification ×100) after 24 h.

## Transwell

SKOV3 cells suspending in 200 μL serum-free medium were implanted in transwell chambers covered with 10% matrigel (BD, Franklin Lakes, NJ, USA). When appropriate cells were filtered to the bottom of the chamber, the cells were fixed with 4% paraformaldehyde and stained with crystal violet. Five random fields per group were photographed under an optical microscope and the number of cells was counted.

## Luciferase activity assay

The luciferase activity was measured using a plate reader (BD bioscience), and normalized to the transfection efficiency by using a Dual Luciferase Reporter Assay Kit (Vazyme, China, Nanjing, No. DL-101-01) as a previous study [10]. All procedures followed the manufacturers’ instructions. All plasmids were constructed by Life Technologies Corporation (Carlsbad, CA) .

## Chromatin immunoprecipitation assay

The chromatin immunoprecipitation (ChIP) assay was performed according to the instructions provided with the ChIP assay kit (Millipore, Darmstadt, Germany). 10% of the chromatin was saved to act as the input control and remainder diluted in CHIP dilution buffer. The diluted chromatin was incubated with 5 μL anti‐SREBF2 antibody or normal immunoglobulin G (IgG). The expression was detected by RT-qPCR. ChIP data were shown as the percentage of the input normalized to control purifications.

## Co-immunoprecipitation (co-IP)

Cells were harvested, and an appropriate volume of lysis buffer (containing protease inhibitor) was added to each sample. The cells were lysed on ice (4°C) for 30 min, after which the supernatants were harvested by centrifugation for 30 min at 300 g. The lysate was added to the cell lysate containing 1 μg of the corresponding antibody. The samples were incubated overnight at 4°C. Next, 10 μl protein A agarose beads (washed with lysate) were added to the aforementioned cell lysates, and slowly shaken at 4°C for 2–4 h to encourage antibody/bead coupling. Following immunoprecipitation, the coupled samples were centrifuged at 350 g (4°C) for 30 min, and the supernatant was discarded. Finally, 15 μl SDS (2X) was added, and the samples were boiled for 5 min prior to western blot analysis.

## Animals and xenografts

Nude female BALB/c mice (6-weeks-old) were obtained from Shanghai SLAC Laboratory Animal Co., Ltd. (Shanghai, China) and were weighed, coded, and randomly assigned to experimental groups. Each group had three mice. All experiments were approved and performed according to the guidelines of the Ethics Committee of the Affiliated Huaian No.1 People’s Hospital of Nanjing Medical University, conformed to the Principles of Laboratory Animal Care (National Society for Medical Research). Tumor formation was assessed in nude mice. The nude mice were divided into shNC, shSREBF2-1, shSREBF2-1+ OE-NC and shSREBF2- 1+ OE-PRSS8. SKVO3 cells (2 × 10^6^) were infected with negative control (NC) or shSREBF2 or overexpression PRSS8. Infected cells were subcutaneously injected into the flanks of 6-week-old male nude mice to induce tumor formation. Tumor diameters were measured at regular intervals every 3 days.

## Statistical analysis

The results are shown as mean ± SD. Statistical analysis was performed using SPSS 20.0. Differences were compared using oneway ANOVA. P < 0.05 indicated a statistical difference.

## Results

### PRSS8 was upregulated in ovarian cancer

**Figure 1.**
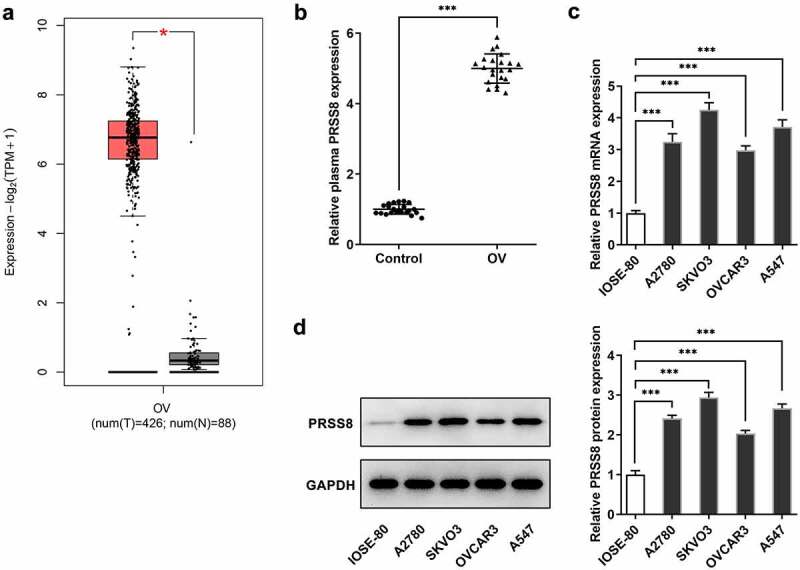
PRSS8 was upregulated in ovarian cancer. A. The GEPIA database predicted the expression of PRSS8 in OC tissues. B. ELISA assay detected the expression of PRSS8 in serum of patients with OC. C. RT-qPCR detected the expression of PRSS8 in cells. D. Western blot detected the expression of PRSS8 in cells.*p < 0.05, ***p < 0.001

The GEPIA database (http://gepia.cancer-pku.cn) predicted that PRSS8 expression was significantly elevated in OC tissues (Figure 1a). We used ELISA to detect the expression of PRSS8 in serum of patients, and we found that the expression of PRSS8 in serum of patients with OC was also significantly higher than that of healthy subjects (Figure 1b). RT-qPCR (Figure 1c) and Western blot (Figure 1d) were used to detect the expression of PRSS8 in OC cell lines, and we found that compared with IOSE-80, the expression of PRSS8 in OC cell lines was also up-regulated at different degrees.

## Knocking down PRSS8 attenuated proliferation, migration, and EMT of OC cells

**Figure 2.**
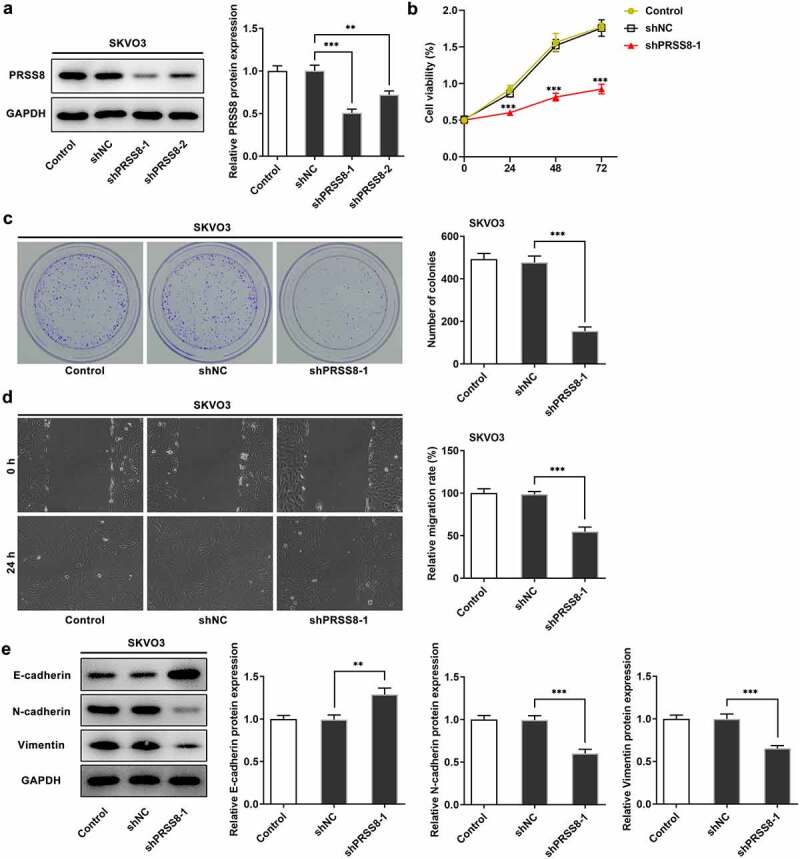
Knocking down PRSS8 attenuated proliferation, migration, and EMT of OC cells. A. Western blot detected the expression of PRSS8 in cells after cell transfection. B. CCK-8 detected the cell viability. C. Clone formation assay detected cell proliferation. D. Wound healing detected the cell migration. E. Western blot detected the expression of EMT-related proteins in cells. **p < 0.01, ***p < 0.001

Next, we examined the effects of PRSS8 on proliferation, invasion and EMT in OC cells. Transfection efficiency was determined by Western blot and we selected shPRSS8-1 for the following experiment (Figure 2a). Inhibition of PRSS8 expression significantly reduced cell viability and proliferation ability (Figure 2b and c). The Wound Healing experiment showed that compared with shNC, the cell migration ability of the shPRSS8 group was significantly reduced (Figure 2d). Western blot was used to detect the expression of EMT-related proteins. Compared with the control group, the expression of E-cadherin was increased in the shPRSS8-1 group, while the expression of N-cadherin and Vimentin was decreased (Figure 2e).

## SREBF2 transcriptional regulated PRSS8

**Figure 3.**
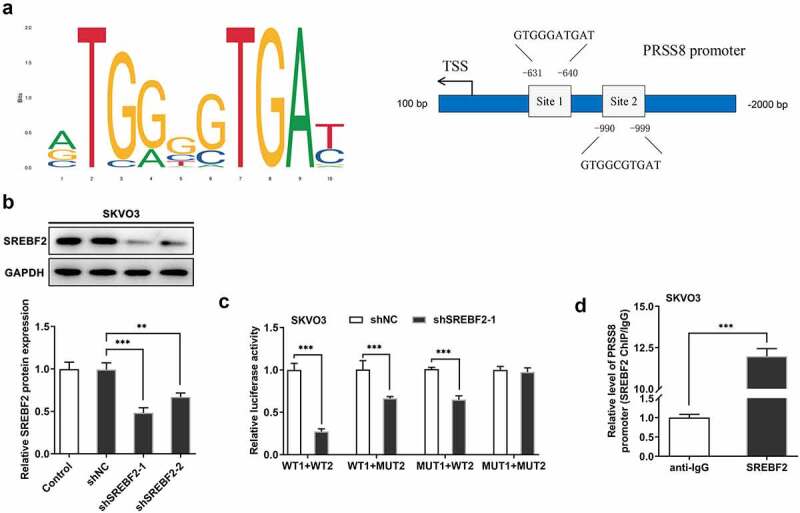
SREBF2 transcriptional regulated PRSS8. A. JASPAR database predicted binding sites of SREBF2 and PRSS8 promoters (Site1 and Site2). B. Western blot detected the expression of SREBF2 in cells after cell transfection. C. The luciferase reporter gene validated the binding between SREBF2 and PRSS8. WT: wild-type PRSS8, MUT: Mutant PPRSS8, 1:site1, 2: site2. D. ChIP test was used to detect the binding relationship between SREBF2 and PRSS8. **p < 0.01, ***p < 0.001

JASPAR database was used to predict that SREBF2 and PRSS8 promoters had binding sites (Site1 and Site2) (Figure 3a). Cell transfection technology inhibited the expression of SREBF2, and the cells were divided into Control, shNC, shSREBF2-1, and shSREBF2-2 groups. Compared with shNC, inhibition of SREBF2-1 significantly decreased PRSS8 expression (Figure 3b). In addition, we verified the targeted binding between SREBF2 and PRSS8 by using luciferase report assay (Figure 3c) and ChIP (Figure 3d) experiments.

## Knocking down SREBF2 reduced PRSS8 and thus inhibited SCNN1A expression

**Figure 4.**
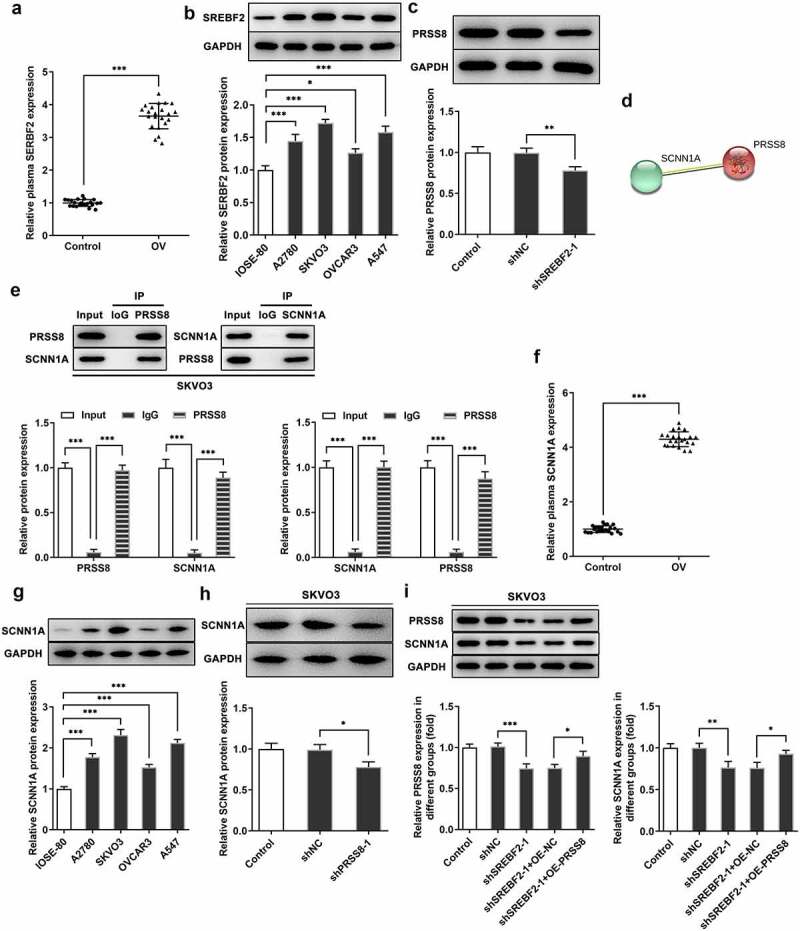
Knocking down SREBF2 reduced PRSS8 and thus inhibited SCNN1A expression. A. ELISA assay detected the expression of SREBF2 in serum of patients with OC. B. Western blot detected the expression of SREBF2 in cells. C. Western blot detected the expression of PRSS8 in cells after cell transfection. D. The String website predicted that PRSS8-targeted regulated SCNN1A expression. E. IP assay detected that PRSS8 binds to SCNN1A. E. The luciferase reporter gene validated the binding between SCNN1A and PRSS8. F. ELISA assay detected the expression of SREBF2 in serum of patients with OC. G. Western blot detected the expression of SCNN1A in cells. C. Western blot detected the expression of SCNN1A in cells after cell transfection. I. Western blot detected the expression of PRSS8 and SCNN1A in cells after cell transfection. *p < 0.05, **p < 0.01, ***p < 0.001

The expression of SREBF2 in the plasma of OC patients and cells was elevated (Figure 4a and b). Then we knocked down the expression of SREBF2 in SKVO3 cells. Western blot was used to detect the expression of PRSS8 (Figure 4c). We predicted by the STRING website that PRSS8 could regulate the expression of SCNN1A (Figure 4d). We found that the expression of SCNN1A in cells was down-regulated after immunoprecipitation of PRSS8 expression and the expression of PRSS8 was down-regulated after immunoprecipitation of SCNN1A. Co-IP experiments verified the binding relationship between PRSS8 and SCNN1A. (Figure 4e). In addition, we found that the expression of SCNN1A in the plasma of OC patients and cells was also elevated (figure 4f and g). After knockdown of PRSS8 expression in SKOV3 cells, the expression of SCNN1A in cells was inhibited (Figure 4h). Then we divided the cells into Control, shNC, shSREBF2-1, shSREBF2-1+ OE-NC and shSREBF2-1+ OE-PRSS8 groups, and we found that after inhibiting the expression of SREBF2 in cells, the expression of PRSS8 and SCNN1A decreased. Further overexpression of PRSS8 increased the expression of PRSS8, and the expression of SCNN1A was reversed (Figure 4i). These results suggest that inhibition of PRSS8 expression in ovarian cancer cells can target and inhibit the expression of SCNN1A.

## Knocking down SREBF2 reduced the proliferation, migration and EMT of OC cells by reducing PRSS8 and thereby inhibiting SCNN1A expression

**Figure 5.**
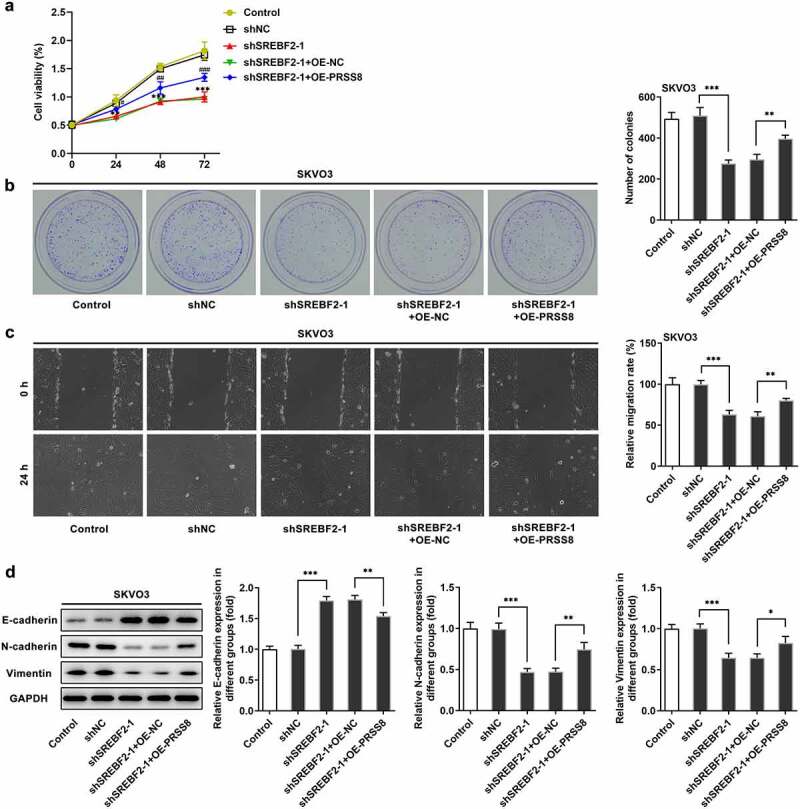
Knocking down SREBF2 reduced the proliferation, migration and EMT of OC cells by reducing PRSS8 and thereby inhibiting SCNN1A expression. A. CCK-8 detected the cell viability. B. Clone formation assay detected cell proliferation. C. Wound healing detected the cell migration. D. Western blot detected the expression of EMT-related proteins in cells. *p < 0.05, **p < 0.01, ***p < 0.001

To further examine the mechanism of action, the cells were divided into control, shNC, shSREBF2-1, shSREBF2-1+ OE-NC and shSREBF2-1+ OE-PRSS8 groups. CCK-8 and colony formation experiment results showed that cell proliferation rate of shSREBF2-1+ OE-PRSS8 group was increased compared with shSREBF2-1+ OE-NC group (Figure 5a and b). The wound healing results showed the same trend as cell proliferation (Figure 5c). Western blot showed that compared with shSREBF2-1+ OE-NC, the expression of E-cadherin in sh SREBF2-1+ OE-PRSS8 group decreased, and the expression of N-cadherin and Vimentin increased (Figure 5d).

## Knocking down SREBF2 inhibited tumor tissue growth in OC mice by reducing PRSS8

**Figure 6.**
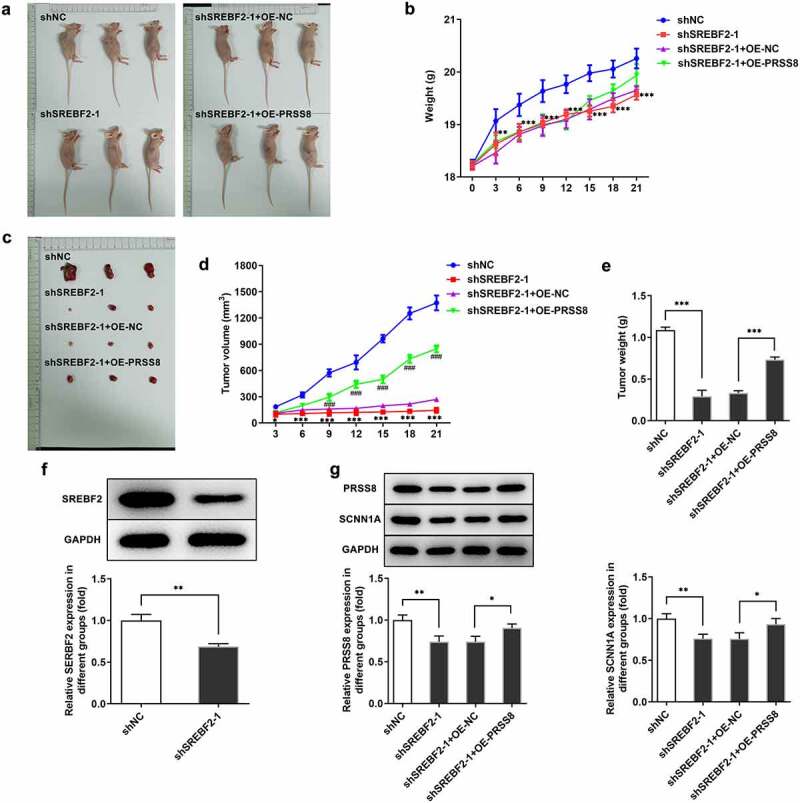
Knocking down SREBF2 inhibited tumor tissue growth in OC mice by reducing PRSS8. A. Photos of nude mice. B. Body weight statistics of mice. C. Tumor tissue photographs. D. Statistics of tumor tissue size. E. Statistical diagram of tumor tissue weight. F. Western blot detected the expression of SREBF2 in cells. G. Western blot detected the expression of PRSS8 and SCNN1A. *p < 0.05, **p < 0.01, ***p < 0.001

In vivo tests (Figure 6a and b) showed the pictures and body weight statistics of tumor-bearing mice. Observation from tumor tissues of mice showed that the tumor tissues of mice with low SREBF2 expression were significantly reduced, while those mice with further overexpression of PRSS8 showed an increased tendency (Figure 6c, d and e). Western blot was used to detect the expressions of SREBF2, PRSS8 and SCNN1A in tumor tissues. Compared with the shNC group, the expressions of SREBF2, PRSS8 and SCNN1A in the shSREBF2-1 group were significantly decreased (figure 6f). After further overexpression of PRSS8, the expression of PRSS8 in tissues was increased and the expression of SCNN1A was reversed (Figure 6g).

## Discussion

PRSS8 is a potential biomarker for early detection of OC [3,11]. The finding in our experiment that the expression of PRSS8 was significantly increased in the plasma of OC patients and OC cell lines was consistent with that concluded by Ayala Tamir et al [3]. Notably, we found that knockdown of PRSS8 expression in OC cells significantly reduced cell proliferation, migration, and EMT capacity, suggesting the role of PRSS8 as an oncogenic molecule in OC. However, controversy emerged to indicate that PRSS8 can act as a tumor suppressor in other cancers. For example, PRSS8 can inhibit the carcinogenesis and metastasis of colorectal cancer [12]. PRSS8 down-regulated and inhibited the growth and metastasis of hepatocellular carcinoma cells [13]. From the above clues, we speculated that PRSS8 might play different regulatory roles in tumors, depending on the specific type of tumor cells or tissues.

We further explored the upstream mechanism of the effects of PRSS8 on the proliferation, invasion and EMT of OC cells, and predicted that SREBF2 was a transcription factor of PRSS8 through TRRUST website. After verification by JASPAR, the binding site between the two was obtained, with regulatory relationship proved through a series of experiments. SREBP2 acted as a transcription factor that regulated genes that encode key proteins involved in cholesterol biosynthesis and low density lipoprotein removal pathways [14]. Combining SREBP2 inhibitors with statins can inhibit the activity of OC cell lines and promote cell apoptosis [15]. In addition, it has been reported that SREBP-2 silencing can up-regulate the expression of CYR61 in osteosarcoma cells, thus reversing the effects of lovastatin on cell invasion and EMT-related proteins [16]. In our experiment, we found that knocking down the expression of SREBF2 can reduce the proliferation, migration and ETM of OC cell lines by downregulating the expression of PRSS8.

By functional experiments, we found that the expression of PRSS8 downstream gene SCNN1A was abnormally elevated in OC, whose binding with SCNN1A was then predicted by the STRING website. Furthermore, as presented by our experiments, knocking down SREBF2 reduced PRSS8 and thus inhibited the expression of SCNN1A, which precisely illustrated the regulatory role of SREBF2. In addition, upregulation of SCNN1A has been reported to promote the proliferation, migration, and poor prognosis of OC cells by regulating EMT [17]. Experimental results showed that SREBF2 knockdown inhibited the expression of SCNN1A by reducing PRSS8, thereby reducing the proliferation, migration and EMT of OC. These results suggest that SCNN1A participates in the enhancement of OC development.

## Conclusion

Overall, we confirmed that SREBF2 can activate the PRSS8/SCNN1A axis to accelerate the cell proliferation, migration and EMT of OC.

## Research highlights

SREBF2 plays a role in OC by regulating the PRSS8/SCNN1A axis

Knocking down SREBF2 reduced the proliferation, migration and EMT of OC cells

Knocking down SREBF2 inhibited tumor growth in OC mice
